# Determinants of choice of skilled antenatal care service providers in Ghana: analysis of demographic and health survey

**DOI:** 10.1186/s40748-018-0082-4

**Published:** 2018-07-11

**Authors:** Kwamena Sekyi Dickson, Eugene Kofuor Maafo Darteh, Akwasi Kumi-Kyereme, Bright Opoku Ahinkorah

**Affiliations:** 1 Department of Population and Health, University of Cape Coast, Cape Coast, Ghana; 2 Department of Health, Physical Education and Recreation, University of Cape Coast, Cape Coast, Ghana

**Keywords:** Skilled providers, Antenatal care services, Utilisation, Women, Ghana

## Abstract

**Background:**

The International Safe Motherhood initiative provides a focus for programmes and research to improve maternal health in low – income countries. Antenatal care is one of the key pillars of the initiative. This study sought to examine the association between background characteristics and choice of skilled providers of antenatal care services in Ghana.

**Methods:**

The study used data from the six rounds of the Ghana Demographic and Health Survey (GDHS). Binary logistic regression models were applied to analyse the data.

**Results:**

Results show that the proportion of women who received antenatal care (ANC) services from skilled providers improved over the period. Also, women with secondary education (OR = 1.42, CI = 1.07–1.88), richest wealth status (OR = 5.10, CI = 2.28–11.85) were more likely to utilise antenatal care services from skilled providers. Whereas women from rural areas (OR = 0.55, CI = 0.41–0.74), with four births or more (OR = 0.55, CI = 0.36–0.85) and from the northern ethnic group were less likely to utilise antenatal care services from skilled providers.

**Conclusion:**

Choice of skilled providers of antenatal care services were predicted by some predisposing factors including education, ethnicity, and ecological zone. Also enabling factors such as wealth status, residence and the need for care factor, parity predicted choice of skilled providers of antenatal care services. Women with secondary or higher education, those within richer and richest wealth status, those from forest zone are more likely to utilise the services of skilled providers during their antenatal care visits. Whereas women from rural areas, those with four births or more and those with the northern ethnic group were more likely to utilise ANC service from unskilled providers. The Ghana Health Service and Ministry of Health should encourage women in rural areas to utilise antenatal care services from skilled providers through social and behaviour change communication campaigns.

## Background

Pregnancy is potentially risky for all women worldwide and millions of women who survive this, experience a form of illness and/or disability related to pregnancy and childbirth [1]. Around the world, complications due to pregnancy and childbearing result in about 830 deaths among women every day and about 303,000 women died during and after pregnancy and childbirth between 1990 and 2015 [2]. In developing countries, maternal mortality ratio in 2015 was 239 per 100,000 live births compared to 12 per 100,000 live births in developed countries [2]. In Africa, dying from complications from pregnancy-related causes during a woman’s’ lifetime is 1 in 40 compared to 1 in 3300 in Europe and 1 in 190 globally [3]. Ninety-nine percent of these deaths occur in developing countries which can be avoided; as the necessary interventions already exist [4]. In sub-Saharan Africa, several countries halved their maternal mortality rates since 1990 [4]. In other regions like Asia and North Africa, the rates of maternal mortality are even much lower, resulting in a 2.3% decline in the global maternal mortality ratio [5]. However, in Ghana, maternal mortality ratio increased from 173 in 2011 [6] to 380 in 2014 [4].

The health of women during pregnancy became an immense concern in the early 1980s due to the high maternal mortality ratios [7]. This is because the care given to women during pregnancy provides interventions that enhance maternal health and survival during the period immediately before and after childbirth [8]. This led to the commencement of the safe motherhood programme. The International Safe Motherhood initiation was launched in Nairobi, Kenya in 1987 and provided a focus for programmes and research concerned with the improvement of maternal health in low – income countries. The safe motherhood programme provides awareness, educational support for the women on screening programs and identifies the problems that make the pregnancy high risk [9]. Antenatal care was one of the key pillars of the safe motherhood [7].

Antenatal Care (ANC) Service has been identified by the World Health Organisation (WHO) as an indicator of maternal health that could be used to help reduce maternal mortality ratios [10]. It is a type of preventative care with the aim of providing regular check-ups that offer doctors or midwives the opportunity to treat and prevent potential health problems throughout the course of the pregnancy while promoting healthy lifestyles that benefit both mother and child [11]. It provides pregnant women and their families with advice and information on health promotion, and preventive health services including the management of a healthy pregnancy, safe childbirth, nutritional support, early exclusive breastfeeding and encouraging assisted delivery [12, 13]. The ultimate aim of ANC is to achieve healthy babies and healthy mothers at the end of pregnancy [14].

The World Health Organisation (WHO) as part of its principles underlying antenatal care advocates that antenatal care should be provided by a health care provider - a skilled attendant. A skilled attendant is explained by WHO as a qualified health professional such as a midwife, doctor or nurse who is trained and educated with expertise to manage normal pregnancies and identify and provide referral for complex problems in women and newborn [15]. Globally, nine out of every ten pregnant women access antenatal care from skilled personnel at least once, only six out of ten access at least four antenatal visits from a skilled provider. In regions where the rates of maternal mortality are high, such as sub–Saharan Africa even fewer women obtain at least four antenatal visits from skilled providers - 49% which is less than the global average [16]. There is also evidence that some pregnant women do not utilise antenatal care services from any provider [17–22]. What factors influence pregnant women to utilise antenatal care from skilled providers?

Using the health care service utilisation model, the current study examines the factors that influence antenatal care services utilisation from skilled providers in Ghana over the period from 1988 to 2014. It contributes to the discourse on skilled providers of antenatal care services in Ghana by examining the factors that influence the use of skilled providers in antenatal care services in Ghana.

### Conceptual framework

**Fig. 1:**
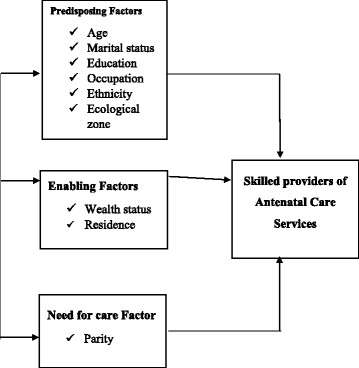
Health service utilisation model. Predisposing factors, enabling factors, need for care factors, Skilled providers of antenatal care

The Health Care Services Utilisation Model is a behavioural model proposed by Anderson in the 1960s to explain the conditions that either promote or hinder the utilisation of health care services [23]. This model identified three main conditions or factors that influence an individual to or not use a health care service as in Fig. 1. These factors are the predisposing factors; enabling factors and need for care factors.

Predisposing factors refer to the demographic, social structure and health belief characteristics of an individual. The demographic characteristics of the individual affect an individual’s decision to use or not use a health care service. The characteristics may include the sex of the individual, the age, and the marital status of the individual. Social Structure consists of education, ethnicity and occupation and health belief factors consist of values, attitudes of health care service providers, and knowledge about health.

Enabling factors are the resources or means that are available to an individual to seek health care services. Enabling factors are measured at the household level and include the availability of income and the community level they include the availability and location of health care facilities in the community. Need for care factors refer to how an individual’s perceive their own general health and functional condition, as well as their familiarity with the signs and symptoms of ill health, agony and concerns about their health [24].

The strength of the model lies in the fact that it looks at the direct factors that lead to the utilisation of health care services. Specifically, it looks at the individual characteristics and the resources or means available to seek health care services. Amid the strength of the health care service utilisation model is that the model embraces both material and structural factors which are scarcely taken into consideration in the social psychological model [25]. The model has been used to compare childhood morbidity and health service utilisation across six countries using the Demographic and Health Survey [26].

The variables used as predisposing factors for the study were age, marital status, education, occupation, ethnicity and ecological zone. Wealth status and residence were also used as enabling factors and parity was used as need for care factor.

## Methods

### Data sources

The Ghana Demographic and Health Survey (GDHS) 1988, 1993, 1998, 2003, 2008, and 2014 used standard DHS model questionnaire developed by the Measure DHS programme [27–32]. The Ghana Demographic and Health Survey is a nationwide survey which covers all ten regions and is conducted every 5 years. The survey is done by the Ghana Statistical Service and the Ghana Health Service with ICF International giving technical support for the survey through MEASURE DHS. The GDHS concentrates on child and maternal health and is designed to offer adequate data to monitor the population and health situation in Ghana. The survey gathers data on various demographic and health issues including fertility, contraceptive use, child health, nutrition, malaria, HIV and AIDS, family planning, health insurance and maternal health; antenatal care, delivery care and post-natal care. For the purpose of the study women with birth history were currently pregnant or has given birth up to 5 years before the survey, thus, 4294 women in 2014, 2131 women in 2008, 2734 women in 2003, 2374 women in 1998, 1974 women in 1998 and 2701 women in 1988 [27–32]. Permission to use the data set was given by the MEASURE DHS following the assessment of a concept note.

### Description and definition of variables

The study used skilled providers of antenatal care (ANC) services as the outcome variable. The outcome variable was derived from the response to the question “did you see anyone for antenatal care for this pregnancy? If YES: whom did you see?” Responses were categorised under Health Personnel and Other Person. Health Personnel included Doctor, Nurse, Nurse/Midwife, Auxiliary Midwife, Community Health Nurse/Officer. Other Person also consisted of Traditional Birth Attendant (TBA), Traditional Health Volunteer, Village Health Volunteer, Other. For the purpose of the study, skilled providers were services provided by: Doctor, Nurse, Auxiliary Midwife, Nurse/Midwife.

The study made use of nine independent variables, these were; maternal age, marital status, educational Level, residence, wealth status, ethnicity, occupation, parity (Birth order), Ecological zone. Maternal Age was categorized into 7 age groups, thus, 15–19, 20–24, 25–29, 30–34, 35–39, 40–44, 45–49. Marital status was originally captured as Never married, Married, Living together, Widowed, Divorced and Not living together but was recoded as Single (Never married, Widowed, Divorced, Not living together), Married and Cohabitation (Living together). Educational Level was classified into four categories: No education, Primary education, Secondary education and Higher education. Type of Residence coded as Urban or Rural. Wealth Quintile was categorised in Poorest, Poorer, Middle, Richer and Richest.

Ethnicity was recoded as Akan (Asante, Akwapim, Fante, and other Akan), Ga– Adangbe, Ewe, Northern Ethnic Groups (guan, Mole–Dagbani, grussi, gruma, hausa, dagarti) and Other. Occupation was captured as not working and working. Parity (birth order) was captioned from a question that measured if respondents have ever given birth. Responses were categorised as Zero (prior to current pregnancy) one birth, two births, three births and four births or more.

The ecological zone was originally coded as Western, Central, Greater Accra, Volta, Eastern, Ashanti, Brong–Ahafo, Northern, Upper East, and Upper west. Region of residence were re–coded to capture the general ecological zones as follows: Northern, Upper East and Upper West regions were re - coded as the ‘Savannah zone’; the Brong–Ahafo, Ashanti and Eastern regions were designated as the ‘Forest zone’; while the Western, Central, Greater Accra and Volta regions were coded as the ‘Coastal zone’.

### Data analysis

The statistical software STATA version 13 was used to process the data. Some variables were recoded and renamed so that they would be consistent across all the rounds and all results were weighted. Univariate and multivariate were carried out. The outcome variable, skilled providers, were coded 0 = No and 1 = Yes. A discrete choice model was employed to show how the independent variables are related to the dependent variable. Precisely, the binary logistic regression was employed given that the model is best fit for dichotomous variables and its ability to predict on a mixture of continuous and categorical variables. The binary logistic regression is based on the assumption that the dependent variable should be dichotomous in nature and the data should not have any outlier.

## Results

Utilisation of the services of skilled ANC provider increased from 45% in 1988 to 88% in 2014.The age of respondents ranged from 15 to 49 years. The mean age of the respondents was 28.6 years in 1988, and about 30 years in 2014. Respondents were predominantly aged 20–29 years. For instance, in 1988, about 40% of the sampled women were in the 20–24 age group and about 41% in 25–29 age group years (see Table 1). The highest proportion of the respondents were from the coastal zone in all the years with the exception of 2003 and 2008, where most of the respondents were predominantly from the forest zone. Majority of the urban respondents used the services of skilled ANC provider- about 75% in 1988 and 96% in 2014 respectively.

**Table 1:** Background Characteristics and choice of skilled providers of ANC

Background characteristic	Years					
	1988	1993	1998	2003	2008	2014
	*n* = 2701	*n* = 1974	*n* = 2374	*n* = 2743	*n* = 2131	*n* = 4294
National	45.0	60.6	68.7	90.8	84.9	87.7
Age						
15–19	38.9	62.8	74.5	91.9	81.0	94.0
20–24	40.4	63.3	73.7	93.9	84.2	89.2
25–29	41.3	61.9	72.9	92.4	90.2	91.3
30–34	50.3	61.4	71.2	91.5	88.2	91.5
35–39	48.7	57.7	69.4	92.2	87.8	91.2
40–44	53.1	53.9	63.9	88.4	81.5	89.6
45–49	49.1	37.5	51.9	89.9	82.8	77.3
Ecological Zone						
Coastal Zone	48.9	66.6	75.4	93.6	87.1	91.7
Forest Zone	38.1	62.0	70.1	94.6	93.5	95.6
Savannah zone	52.3	47.9	59.2	84.7	74.5	77.9
Place of Residence						
Urban	43.1	75.4	83.1	97.6	94.1	95.7
Rural	45.7	54.7	66.4	88.8	81.9	86.0
Level of Education						
No education	48.8	50.8	63.2	86.2	77.7	81.4
Primary	41.3	65.1	75.8	92.3	85.9	89.7
Secondary	47.6	85.7	74.6	97.4	93.1	94.9
Higher	58.8	93.3	84.8	100.0	99.3	98.8
Wealth Status						
Poorest	–	64.3	66.7	83.4	71.9	78.2
Poorer	–	57.9	70.9	91.3	83.4	88.0
Middle	–	58.0	70.1	94.9	92.1	93.0
Richer	–	59.5	71.1	95.1	94.1	96.1
Richest	–	62.8	72.8	98.7	97.5	98.7
Marital Status						
Single	53.8	57.6	71.7	91.1	87.7	92.0
Married	43.3	60.9	70.6	91.8	86.7	90.8
Cohabitation	51.2	60.7	71.1	94.6	86.4	88.7
Parity						
One birth	42.7	68.8	78.0	94.7	90.5	94.1
Two births	42.0	62.7	72.5	94.0	88.5	92.6
Three births	44.7	63.1	72.8	92.4	88.4	91.4
Four or more births	47.1	54.9	65.5	89.5	83.2	87.0
Ethnicity						
Akan	41.5	63.0	73.9	94.7	93.1	94.3
Ga/Dangme	54.0	80.5	79.9	93.9	92.3	93.5
Ewe	43.7	62.1	73.5	92.6	81.7	92.8
Northern Ethnic Group	48.5	51.5	60.1	86.5	79.2	83.1
Other	52.1	62.5	61.2	90.6	82.1	91.9
Occupation						
Not working	44.1	63.2	66.0	90.5	89.4	91.4
Working	45.8	59.9	71.5	92.2	86.5	90.4

Source: Computed from GDHS 1988, 1993, 1998, 2003, 2008 and 2014

The level of education of the respondents who predominantly utilised the services of a skilled ANC provider for the years under review were with higher education. For instance, 59% in 1988; and about 98% in 2014 (see Table 1). Majority of the respondent within the richest wealth status used the services of a skilled provider during their ANC visits over the years under review (see Table 1). The highest proportion of women with one birth utilized the services of skilled providers during their ANC visits over the years under review. For instance, about 69% in 1993, 78% in 1998, 94% in 2003, 91% in 2008 and 94% in 2014 (see Table 1). With the exception of 1988 and 2003, single women dominated among those who use the services of a skilled ANC provider during their ANC visits over the years under review. The highest proportion of the respondents from the Ga/Dangme ethnic group utilised the services of a skilled ANC provider in all the year with the exception of 2003, 2008 and 2014) which was dominated by the women from the Akan ethnic group (see Table 1).

The results suggest that women from the forest zone (OR = 2.78, CI = 2.02–3.83) were more likely to use the ANC services from skilled providers during antenatal care services as compared to those from the coastal zone (see Table 2). While women from Northern ethnic group (OR = 0.63, CI = 0.44–0.92) were less likely to utilise antenatal care services from skilled providers during antenatal care as compared to Akan women.

**Table 2:** Logistic Regression on Choice of Antenatal Care Services Provided by Skilled Providers from 1988 to 2014

Variable	Odds ratio (OR)	Confidence Interval (CI)
Age		
15–19	1.41	0.77–2.59
20–24	Ref	Ref
25–29	1.24	0.89–1.73
30–34	1.31	0.83–1.82
35–39	1.31	0.86–2.02
40–44	1.12	0.70–1.80
45–49	0.83	0.48–1.45
Ecological Zone		
Coastal zone	Ref	Ref
Forest zone	2.78***	2.02–3.83
Savannah zone	1.15	0.79–1.67
Ethnicity		
Akan	Ref	Ref
Ga/Dangme	0.85	0.48–1.53
Ewe	1.06	0.72–1.57
Northern ethnic group	0.63*	0.44–0.92
Other	1.38	0.52–3.66
Level of Education		
No education	Ref	Ref
Primary	1.37*	1.04–1.80
Secondary	1.42*	1.07–1.88
Higher	3.41	0.52–3.66
Occupation		
Not working	Ref	Ref
Working	1.15	0.87–1.50
Marital status		
Single	Ref	Ref
Married	1.28	0.93–1.76
Cohabitation	0.78	0.55–1.11
Wealth status		
Poorest	Ref	Ref
Poorer	1.33*	1.01–1.76
Middle	1.83**	1.27–2.65
Richer	2.03**	1.26–3.26
Richest	5.19***	2.28–11.85
Place of Residence		
Urban	Ref	Ref
Rural	0.55***	0.41–0.74
Parity		
One birth	Ref	Ref
Two births	0.79	0.54–1.16
Three births	0.67*	0.44–1.00
Four births or more	0.55**	0.36–0.85
Survey wave years		
1988	Ref	Ref
1993	5.98***	5.21–6.87
1998	6.13***	5.39–6.99
2003	10.54***	9.12–12.19
2008	7.79***	6.73–9.01
2014	30.4***	25.39–36.43

Source: computed from GDHS 1988, 1993, 1998, 2003, 2008 and 2014

*Ref* reference, *OR* Odds Ratio

*p < 0.10 **p < 0.05 ***p < 0.001

There was a significant relationship between education and utilisation of ANC services received from skilled providers varied. For instance, women with secondary education (OR = 1.34, CI = 1.07–1.88) were more likely to utilise the services of a skilled provider during antenatal care compared to women with no education. With reference to residence, it was observed that women from rural areas (OR = 0.55, CI = 0.41–0.74) were less likely to use the services of a skilled provider during antenatal care as compared to those from the urban areas (see Table 2).

Also, the likelihood of skilled providers providing antenatal care services varied by wealth status and parity. For instance, women with the richest wealth status were more likely to utilise the services of skilled providers (OR = 5.19, CI = 2.28–11.88) (see Table 2) during antenatal care services as compared to women within the poorest wealth status, whiles women with four births or more (OR = 0.55, CI = 0.36–0.85) were also less likely to utilise the services of skilled providers during antenatal care than those with one birth (see Table 2). The likelihood of women receiving antenatal care service from a skilled provider varied from survey wave years (see Table 2). For instance, it was observed that the use of ANC from skilled providers increased in survey wave year 1993 (OR = 5.98, CI = 5.21–6.87); 1998 (OR = 6.13, CI = 5.39–6.99); 2003 (OR = 10.54, CI = 9.12–12.19). Even though the odds decreased in 2008(OR = 7.79, CI = 6.73–9.01), it increased again in 2014 (OR = 30.41, CI = 25.39–36.43).

## Discussion

Using the Health Care Service Utilisation model, the study sought to examine the predictors of skilled providers of antenatal care services in Ghana over the period 1988 to 2014. Predisposing factors such as education, ethnicity and ecological zones had significant relationship with skilled providers of antenatal care services. Also, enabling factors such as residence and wealth status had similar associations. Need for change factor, parity, had significance relationship with skilled providers of antenatal care services.

The study observed that women with secondary or higher education are more likely to receive antenatal care services from skilled providers compared with those with no education. This finding is consistent with findings of other studies [17, 19, 33–35]. The possible explanation for the findings is that women with secondary or higher education may have more awareness about the danger signs and complications of pregnancy and may know the need to receive ANC services from a skilled provider.

As expected, wealth status was found to have a significant relationship with skilled providers of antenatal care services. Women within the middle, richer and richest wealth status were more likely to utilise antenatal care services from skilled providers compared to women within the poorest wealth status. This is in line with the findings of Atunah - Jay, Pettingell, Ohene, Oakes, & Borowsky [20], Ganle et al. [19] and Wang, Alva, Wang and Fort [35] found that women with richest wealth status were more likely to receive ANC services from skilled personnel compared to those from poorest wealth status who utilised ANC services from traditional birth attendants.

Furthermore, women from rural areas were less likely to utilise the services of skilled providers. This is consistent with the findings by Dickson, Darteh and Kumi-Kyereme [17], who also found that women in rural areas were less likely to receive ANC services from doctors and nurses compared to those in urban areas. One reason for the findings could be the urban-rural gap in the distribution of health resources, where those in urban areas are more likely to have access to tertiary health centres compared to those in rural areas. Others reasons could be that women in rural areas prefer home delivery because of lack of health facility near home and being more comfortable at home. A study by [36] also found that reasons for low utilization of ANC services by women in rural areas include walking long distances to access health facilities, lack of midwives, lack of or insufficient items to be used during delivery, long stay and rude health personnel.

The study established that women from the northern ethnic groups were less likely to utilise antenatal care services from skilled providers compared to women from the Akan ethnic group. Cultural beliefs and morals are believed to shape the lives and behaviours of people and this can have influences in their choices of health care provider [37]. Women from minority ethnic group were found not to utilise antenatal care services from skilled provider compared to women from majority ethnic group [38, 39]. Like rural-urban disparity in healthcare utilization by pregnant women in Ghana, the country also experiences differences in access and use of healthcare resources in terms of north-south divide where people in the northern part of the country have low access to healthcare resources including skilled personnel compared to those in the southern part of the country. The inadequate access to healthcare resources could explain why from the northern ethnic groups were less likely to utilise antenatal care services from skilled providers compared to women from the Akan ethnic group, who predominantly live in the southern part of the country.

Our study also observed that parity was associated with receiving antenatal care services from skilled providers. Women with four births or more were less likely to receive ANC services from skilled providers compared to women with parity one. This could be as a result of the experiences and knowledge gained from their previous births hence they may not be willing to go to a skilled provider for antenatal care services. This finding is consistent with Shrestha, Bell and Marais [40], 19Ganle et al. [19] and Wang et al. [35]. Finally, women from forest zone were more likely to utilise ANC services from skilled providers compared to women from the coastal zone. Similar findings were obtained in a study by Dickson, Darteh and Kumi-Kyereme [17], who also found that women from forest zone were more likely to utilise ANC services from nurses compared to those in the coastal zone. The reason for this finding is that the forest zone is mostly made up of more urban than rural communities and most communities in the forest zone have more access to healthcare resources, explaining why the utilization of ANC services in high in those areas.

## Conclusion

The study investigated the factors influencing the choice of skilled providers during antenatal care visits among women in Ghana over the period from 1988 to 2014 using the Health Service Utilisation Model. Choice of skilled providers of antenatal care services were predicted by some predisposing factors including education, ethnicity, and ecological zone. Also enabling factors such as wealth status, residence and the need for care factor, parity. Women with secondary or higher education, those within richer and richest wealth status, those from forest zone are more likely to utilise the services of skilled providers during their antenatal care visits. The Ghana Health Service and Ministry of Health should encourage women in rural areas to utilise antenatal care services from skilled providers through social and behaviour change communication campaigns. This can be achieved by government through providing healthcare resources such as skilled health personnel as well as materials and equipment in health facilities in rural areas that will make pregnant women feel more comfortable accessing antenatal care.

### Data limitations

The Ghana Demographic and Health Survey uses a repeated cross-sectional design and the sample that were used were not carried to all the rounds, chances could be that different set respondents were used for the survey in all the different rounds. Changes in the sample over time may have effects on the results due to inherent characteristics. There is also the limitation that comes with self-reporting made by the mothers’ interview. Questions were based on birth history; women were asked questions about themselves three to 5 years before the round of the survey. This may be affected by recall bias or deliberate misreporting.
